# DESPERATE TIMES CALL FOR DESPERATE MEASURES: GOVERNMENT SPENDING MULTIPLIERS IN HARD TIMES

**DOI:** 10.1111/ecin.12919

**Published:** 2020-07-08

**Authors:** Sokbae Lee, Yuan Liao, Myung Hwan Seo, Youngki Shin

**Affiliations:** ^1^ Department of Economics Columbia University New York NY 10027 USA; ^2^ Research Staff Institute for Fiscal Studies London WC1E 7AE UK; ^3^ Department of Economics Rutgers University New Brunswick NJ 08901 USA; ^4^ Department of Economics Seoul National University Seoul 08826 Republic of Korea; ^5^ Department of Economics McMaster University Hamilton ON L8S 4L8 Canada

## Abstract

We investigate state‐dependent effects of fiscal multipliers and allow for endogenous sample splitting to determine whether the U.S. economy is in a slack state. When the endogenized slack state is estimated as the period of the unemployment rate higher than about 12%, the estimated cumulative multipliers are significantly larger during slack periods than nonslack periods and are above unity. We also examine the possibility of time‐varying regimes of slackness and find that our empirical results are robust under a more flexible framework. Our estimation results point out the importance of the heterogenous effects of fiscal policy and shed light on the prospect of fiscal policy in response to economic shocks from the current COVID‐19 pandemic. *(*JEL *C32, E62, H20, H62)*

**ABBREVIATIONS**
GDP: gross domestic product
MIO: mixed integer optimization

## I INTRODUCTION

The debate over the role of fiscal policy during a recession has recently taken center stage again in macroeconomics. One particular topic that has received substantial attention is whether the multiplier effect of government spending is state‐dependent. On the one hand, in a series of papers, Auerbach and Gorodnichenko (2012, 2013a, 2013b) used data from the United States as well as from the organization for economic cooperation and development countries and provided empirical evidence supporting that the fiscal multiplier might be larger during recessions than expansions. On the other hand, Ramey and Zubairy (2018) constructed new quarterly historical U.S. data and reported that their estimates of the fiscal multipliers were below unity irrespective of the state of the economy.

In this paper, we contribute to this debate by estimating a threshold regression model that determines the states of the economy endogenously. Auerbach and Gorodnichenko (2012) estimated smooth regime‐switching models using a 7 quarter moving average of the output growth rate as the threshold variable. Their primary results relied on a fixed level of intensity of regime switching. Instead of estimating the level of intensity jointly with other parameters in their model, they calibrated the level of intensity so that the U.S. economy spends about 20% of time in a recessionary regime. In Ramey and Zubairy (2018), the baseline results assume that the U.S. economy is in a slack state if the unemployment rate is above 6.5%. To check the baseline results, Ramey and Zubairy (2018) conducted various robustness checks using different thresholds.

To be consistent with the empirical literature, we build on Ramey and Zubairy (2018): we use their dataset and follow their methodology closely. Our main departure from the recent empirical literature is that we split the sample in a data‐dependent way so that the choice of threshold level is determined endogenously. It turns out that the endogenized threshold level of the unemployment rate is estimated at 11.97%, which is much higher than 6.5% adopted in Ramey and Zubairy (2018). Using this new threshold level combined with the same data and specifications as in Ramey and Zubairy (2018), we find that the estimated fiscal multipliers are significantly different between the two states and above unity for the high unemployment state. Specifically, if the threshold level is 6.5%, the estimates of 2‐year integral multipliers are around 0.6 regardless of the state of the economy. However, if the threshold level is 11.97%, the estimates are 1.58 for the high employment state and 0.55 for the low employment state, respectively. If we look at observations used in estimation, there is no period after World War II with the unemployment rate higher than 11.97%. In fact, there is only one timespan of severe slack periods in 1930s. In other words, the period of the Great Depression is isolated from other periods, as an outcome of our estimation procedure. Therefore, our estimation results suggest that (1) the fiscal multiplier can be larger than unity if the slackness of the economy is very severe and that (2) the post‐World War II period does not include the severe slack state and thus, our estimates for the high unemployment state are not applicable to moderate recessions in the post‐WWII period. However, after the outbreak of the COVID‐19 pandemic, the U.S. unemployment rate rose to 14.7% in April 2020.^1^ Therefore, the estimation results in this paper shed light on the prospect of the fiscal policy in response to the current economic shocks. We also examine the possibility of time‐varying regimes of slackness by including a time dummy for the post‐WWII period and find that our empirical results are robust under this more flexible framework. All the computer codes and data files for replication are available at https://github.com/yshin12/llss-rz.

The remainder of the paper is organized as follows. In Section II, we describe the econometric model and present empirical results. In Section III, we give concluding remarks.

## II MODEL AND EMPIRICAL RESULTS

In this section, we give a brief description of the methodology developed by Ramey and Zubairy (2018, RZ hereafter). They consider the state‐dependent local projection method of Jordà (2005). Their baseline regression model for each horizon *h* has the following form (see equation (2) in RZ):

(2.1) $$\begin{array}{ll} & x_{t+h}=I_{t-1}\left(\alpha_{A,h}+\psi_{A,h}\left(L\right)z_{t-1}+\beta_{A,h}{\text{shock}}_{t}\right)\\ & +\left(1-I_{t-1}\right)\left(\alpha_{B,h}+\psi_{B,h}\left(L\right)z_{t-1}+\beta_{B,h}{\text{shock}}_{t}\right)\\ & +\epsilon_{t+h}, \end{array}$$

where *I*_*t*_(·) is a dummy variable denoting the state of the economy, *x*_*t*_ is the variable of interest, *z*_*t*_ is a vector of control variables including GDP, government spending, and lags of the defense news variable, *ψ*(*L*) is a polynomial of order 4 in the lag operator, and *shock*_*t*_ is the defense news variable.

Recall that RZ assume that the economy is in the slack state when the unemployment rate is above 6.5%. We instead adopt a threshold regression model and parameterize *I*_*t*_ = 1{*unemp*_*t*_ > *τ*}, where 1{·} is an indicator function and *unemp* denotes the unemployment rate. In other words, we estimate the model that endogenously determines the slack states that fit the data best. Specifically, we estimate the following model using the least squares (see, e.g., Hansen 2000; Hidalgo, Lee, and Seo 2019):

(2.2) $$\begin{array}{ll} & {\mathrm{GDP}}_{t}=1\left\{{\text{unemp}}_{t-1}>\tau \right\}\\ & \times \left(\alpha_{A}+\psi_{A}\left(L\right)z_{t-1}+\beta_{A}{\text{shock}}_{t}\right)\\ & +1\left\{{\text{unemp}}_{t-1}\leq \tau \right\}\\ & \times \left(\alpha_{B}+\psi_{B}\left(L\right)z_{t-1}+\beta_{B}{\text{shock}}_{t}\right)+\epsilon_{t}. \end{array}$$

To estimate the threshold regression model in (2.2), define the objective function

$$\begin{array}{l} Q_{T}\left(\tau ,\theta \right):=\sum_{t=1}^{T}\left[{\mathrm{GDP}}_{t}-1\left\{{\text{unemp}}_{t-1}>\tau \right\}\right.\\ \times \left(\alpha_{A}+\psi_{A}\left(L\right)z_{t-1}+\beta_{A}{\text{shock}}_{t}\right)\\ \;-1\left\{{\text{unemp}}_{t-1}\leq \tau \right\}\left(\alpha_{B}+\psi_{B}\left(L\right)z_{t-1}\right.\\ {\left.\left.+\beta_{B}{\text{shock}}_{t}\right)\right]}^{2}, \end{array}$$

where *θ* := (*α*_*A*_, *ψ*_*A*_(*L*), *β*_*A*_, *α*_*B*_, *ψ*_*B*_(*L*), *β*_*B*_). Note that the model (2.2) is linear in *θ* conditional on *τ* . Thus, we obtain the (restricted) ordinary least squares estimator $\hat{\theta }\left(\gamma \right)$ easily for any given *γ* . Then, the threshold parameter *γ* can be estimated by minimizing the profiled objective function:

$$\hat{\tau }:=\underset{\tau \in T}{\text{argmin}}Q_{T}^{*}\left(\gamma \right),$$

where $Q_{T}^{*}\left(\gamma \right):=Q_{T}\left(\gamma ,\hat{\theta }\left(\gamma \right)\right)$. To estimate this model, it is necessary to specify the parameter space T for *τ* . We set it to be the interval between the 5 and 95 percentiles of the unemployment rates in the dataset and estimate $\hat{\tau }$ by the grid search method.

In our view, the threshold regression model above provides a natural way to endogenize the level of slackness since there is a change point at *τ* for GDP in the model. Note that the level of the slackness is determined endogenously by fitting the regression model for GDP in (2.2) and then it is imposed in the specification of *I*_*t* − 1_ in (2.1). Considering that both RZ and Auerbach and Gorodnichenko (2012, 2013a, 2013b) determine the criterion for the economic slackness based on the researchers' discretion, it is novel to determine the threshold point endogenously. Furthermore, as we will see in the next section, the endogenous threshold estimate is beyond the range of the values that RZ considered for a robustness check.

In general, estimating the change point *τ* tends to be robust to model misspecification. Specifically, in our context, the local projection argument may imply that the model (2.2) is potentially misspecified; however, it is worthwhile to emphasize that the change‐point estimation tends to be robust against mild misspecification in the regression function employed in each regime, as shown by for example Bai et al. (2008).

Before looking at the estimation results, we briefly describe the dataset adopted in our empirical analysis. RZ constructed new quarterly U.S. data from 1889 to 2015 for their analysis. The main variables include real GDP, real government spending, the unemployment rate, and the defense news series. The real GDP data come from Historical Statistics of the United States for 1889–1928 and from the National Income and Product Accounts from 1929 to 2015. Real government spending is calculated by dividing all federal, state, and local purchases by the GDP deflator. The unemployment rates before 1948 were calculated by interpolating Weir's (1992) series and the NBER Macrohistory database. Finally, the defense news series is constructed by the narrative method of Ramey (2011), which measures changes in the expected present discounted value of government spending. For additional details of the dataset, we refer to Ramey and Zubairy (2018).

### A Endogenous Sample Splitting

**FIGURE 1:**
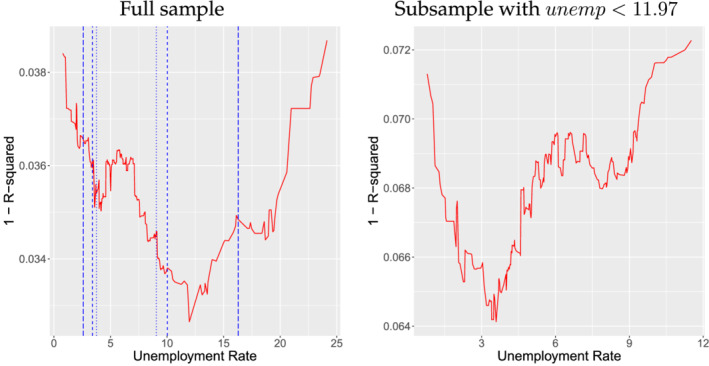
Least Squares Objective Function *Note*: In the left‐hand panel, the long‐dashed vertical lines are the 5 and 95 percentiles of the empirical distribution of the unemployment rate. The dashed vertical lines are the 10 and 90 percentiles and the dotted lines are the 15 and 85 percentiles, respectively.

Using the same dataset constructed by RZ, we obtain $\hat{\tau }=11.97\%$ for the threshold parameter. This estimate is even higher than 8%, which RZ used for their robustness check. To appreciate our estimation result, we plot the profiled least squares objective function (1 − *R* ^2^ ) as a function of *τ* in the left‐panel of Figure 1.

It can be seen that the minimizer is well separated at 11.97%, which gives the graphical verification of $\hat{\tau }$. On the contrary, there is even no local minimum around RZ's threshold value at 6.5%. To check the possibility of the second threshold level below 11.97%, we re‐estimated the model with the subsample for which the unemployment rate is lower than 11.97%. The right‐hand panel indicates that there could be a second threshold around 4%, but not around 6.5%.

**FIGURE 2:**
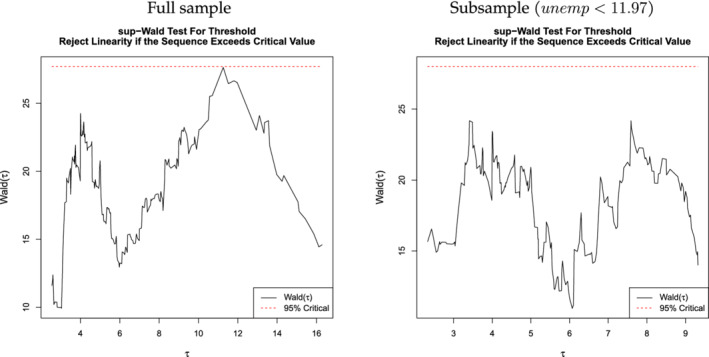
Inference for Multiple Regimes *Note*: The red dashed line denotes the 95% critical value for the existence of the threshold point. In the left panel, we confirm that the Wald test statistic at *τ* = 11.97 is very close to the 95% critical value. In the right panel, we use the subsample and test if there exists an additional threshold point. The result confirms that there is no additional threshold point in the subsample.

We test for the existence of the threshold for the whole sample and for the subsample with *unemp* < 11.97 by adopting the sup‐Wald test in Hansen (1996). Figure 2 gives a graphical summary of the testing results. We set the number of bootstraps to 2,000 and the trimming ratio to 5%. We use the heteroskedasticity‐robust test statistic. The bootstrap *p*‐value for the whole sample is 0.053 and we can reject the null hypothesis of no threshold effect at the 10% significance level. For the subsample with the unemployment rate below 11.97, the bootstrap *p*‐value for the same test is 20.3%. Thus, we conclude that there is mild evidence for the single threshold in the data. Finally, the 95% confidence interval for the threshold variable is (11.97, 13.56).

**FIGURE 3:**
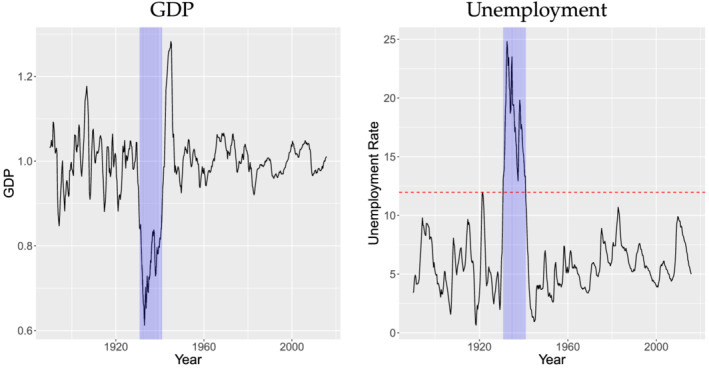
Periods of Slack States over GDP and Unemployment *Note*: *GDP* denotes real per capita GDP divided by trend GDP. The red dashed line in the right panel is the change‐point estimate, $\hat{\tau }=11.97$. The blue shaded area denotes the slack states estimated from the data.

The periods with high unemployment rates are relatively rare. The U.S. economy spent less than 10% of time in the new slack regime defined by 11.97%. The shaded areas in Figure 3 show slack periods over GDP and unemployment rates. There is only one timespan of severe slack periods from 1930Q3 to 1940Q3, namely the Great Depression. We call this new slack periods as severe slack states (“hard times”) compared to moderate slack states in RZ. There is no period after WWII that belongs to the hard times in this dataset. However, the current recession belongs to the hard times, as the unemployment rose to 14.7% in April 2020.

### B State‐Dependent Cumulative Multipliers

**FIGURE 4:**
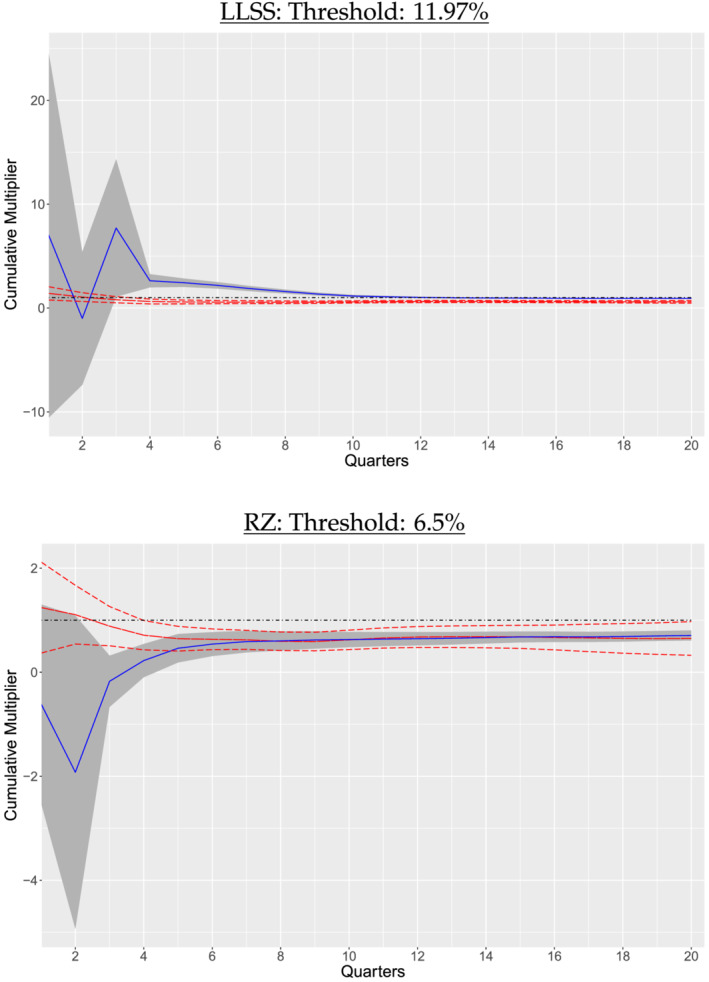
Cumulative Multipliers *Note*: The blue solid line denotes cumulative multipliers for slack states (high unemployment) and the red dashed line for nonslack states (low unemployment). The 95% pointwise confidence bands are also presented along with cumulative multipliers. We also draw a dot‐dashed horizontal line at multiplier = 1.

We now report the estimation results of the cumulative multipliers under endogenous sample splitting. It turns out that the new regime classification produces quite different implications. Following RZ, we adopt the local projection method in Jordà (2005) and use the military news as an instrument. Figure 4 reports the cumulative multiplier over 5 years (20 quarters) in each regime. To make the comparison straightforward, we also show the estimation results of Ramey and Zubairy (2018) next to our results.

**FIGURE 5:**
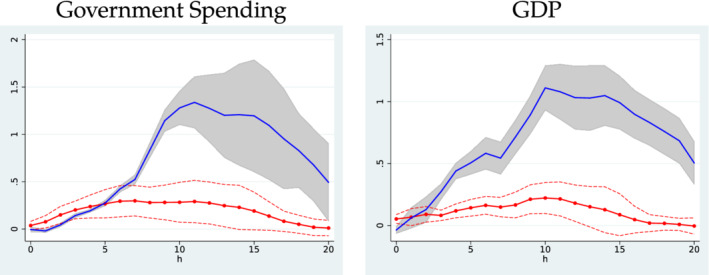
Government Spending and GDP Responses to News Shock *Note*: A news shock is equal to 1% of GDP. The red line with circles denotes the impulse response function in nonslack periods and the blue solid line denotes the same function in slack periods. The related 95% pointwise confidence bands are also provided. The threshold point dividing slack/nonslack periods is $\hat{\tau }=11.97$ estimated from the data.

When the 6.5% threshold is used in classification of slack state (i.e., the moderate slack state), the multipliers in the high‐unemployment state are negative up to 3 quarters and are indistinguishable to those in the low‐unemployment state after 6 quarters. It is counterintuitive to observe that the multipliers are higher for the low unemployment state. On the other hand, if the 11.97% threshold is adopted (i.e., the severe slack state), the multipliers in the high‐unemployment state are mostly positive and largely above those in the low‐unemployment state and are around unity after 10 quarters. In other words, the multipliers are all less than unity in the case of the moderate slack state; however, they are substantially higher in the case of the severe slack state. These results are robust to the choice of the instrumental variable. As additional empirical results, Figure 5 depicts the impulse response functions in non‐slack and slack periods, respectively. Both government spending and GDP responses are much higher in slack periods.

In Table 1, we report the 2‐year and 4‐year cumulative multipliers when we use the military news, Blanchard and Perotti (2002) shock, and the combined variable of these two as an instrument, respectively. The basic implication does not change. The estimates of the 2‐year multiplier vary from 1.58 to 2.21 and the 4‐year multipliers are around 1. The main implication from our empirical results is that fiscal multipliers can be significantly larger during severe recessions than in normal periods.

**TABLE 1:** Estimates of Cumulative Multipliers

	High Unemployment	Low Unemployment	*p*‐value for difference in multipliers
*Panel A: Threshold at 11.97%*			
Military news shock			
2 year integral	1.58	0.55	0.000
	(0.099)	(0.064)	
4 year integral	0.94	0.61	0.000
	(0.017)	(0.050)	
Blanchard–Perotti shock			
2 year integral	1.65	0.34	0.005
	(0.425)	(0.105)	
4 year integral	1.23	0.40	0.000
	(0.130)	(0.104)	
Combined			
2 year integral	2.21	0.35	0.000
	(0.406)	(0.092)	
4 year integral	1.11	0.46	0.000
	(0.108)	(0.086)	
*Panel B. Threshold at 6.5%*			
Military news shock			
2 year integral	0.60	0.59	0.954
	(0.095)	(0.091)	
4 year integral	0.68	0.67	0.924
	(0.052)	(0.121)	
Blanchard–Perotti shock			
2 year integral	0.68	0.30	0.005
	(0.102)	(0.111)	
4 year integral	0.77	0.35	0.001
	(0.075)	(0.107)	
Combined			
2 year integral	0.62	0.33	0.099
	(0.098)	(0.110)	
4 year integral	0.68	0.39	0.021
	(0.052)	(0.110)	

*Note*: The *p*‐values for difference in multipliers are calculated by the HAC‐robust *p*‐values in Newey and West (1987). Panel A is based on our threshold estimate (11.97%). Panel B comes from Ramey and Zubairy (2018) where the threshold point (6.5%) is chosen by the authors.

We illustrate the difference between our results and those in RZ by comparing the effects of the COVID‐19 stimulus package. The COVID‐19 pandemic and the following economic lockdown increased the U.S. unemployment rate up to 14.7% in April 2020. This is the highest unemployment rate since World War II. To mitigate the economic hardship, the U.S. congress has passed the COVID‐19 stimulus package (the CARES act) whose total amount is 2 trillion dollars. In Table 2, we report the difference of the estimated multiyear integral effects of the stimulus package when we use the multipliers in this paper and those in RZ. We assume that 25% of the total amount (500 billion dollars) will be spent in the immediate quarter and use the cumulative multiplier estimates based on the military news shock. Two approaches provide quite different results of the policy effect. Over 2 years, the difference between the two estimates is 490 billion dollars. The gap decreases over time but it is still 70 billion dollars after 5 years. Therefore, we conclude that the endogenous threshold estimate gives quite different results of the fiscal policy effect, especially when the slackness of the economy is severe.

**TABLE 2:** GDP Increases Caused by the COVID‐19 Stimulus Package (in $ bn)

	LLSS (Threshold at 11.97%)	RZ (Threshold at 6.5%)	Difference
2 year integral	790	300	490
3 year integral	510	355	155
4 year integral	470	340	130
5 year integral	465	395	70

*Note*: The estimates denote the increased cumulate GDP when the U.S. government spends 500 billion dollars in the period of high unemployment (14.7%). Military news shocks are used as an instrument.

### C Possibly Time‐Varying Regimes

In this subsection, we explore the possibility of time‐varying regimes of slackness. One might be worried that the U.S. economy changed after WWII such that the level of slackness changed from the pre‐WWII period to the post‐WWII period. To deal with this issue, we extend the endogenous sample splitting to the following specification:

$$I_{t-1}=1\left\{{\text{unemp}}_{t-1}+\tau_{1}d_{t-1}-\tau_{0}>0\right\},$$

where *d*_*t*_ = 1 if *t* is greater than or equal to 1945Q4. The resulting regression model has the following form:

$$\begin{array}{ll} & {\mathrm{GDP}}_{t}=1\left\{{\text{unemp}}_{t-1}+\tau_{1}d_{t-1}-\tau_{0}>0\right\}\\ & \times \left(\alpha_{A}+\psi_{A}\left(L\right)z_{t-1}+\beta_{A}{\text{shock}}_{t}\right)\\ & +1\left\{{\text{unemp}}_{t-1}+\tau_{1}d_{t-1}-\tau_{0}\leq 0\right\}\\ & \times \left(\alpha_{B}+\psi_{B}\left(L\right)z_{t-1}+\beta_{B}{\text{shock}}_{t}\right)+\epsilon_{t}. \end{array}$$

To estimate this model, we need to optimize the least squares objective function with respect to unknown parameters jointly. The parameters could be estimated through the profiling method as explained in Section II. Specifically, one may first estimate the slope parameters *θ* := (*θ*_*A*_, *θ*_*B*_) = (*α*_*A*_, *ψ*_*A*_, *β*_*A*_, *α*_*B*_, *ψ*_*B*_, *β*_*B*_) given *τ* := (*τ*_0_, *τ*_1_) and then optimize the profiled objective function over *τ* by the two‐dimensional grid search.

We adopt more efficient computational algorithms developed in our previous work (Lee et al. 2018) with the aid of mixed integer optimization (MIO). To explain the algorithm, we first define some notation: *y*_*t*_ := *DGP*_*t*_ , *f*_*t*_ := (*unemp*_*t* − 1_, *d*_*t* − 1_, −1), and *x*_*t*_ := (1, *z*_*t* − 1_, *shock*_*t*_). Then, the least squares estimator can be written as

(2.3) $$\begin{array}{cc} & \left(\hat{\tau },{\hat{\theta }}_{B},\hat{\delta }\right):=\underset{\tau ,\theta_{B},\delta }{\text{argmin}}\sum_{t=1}^{T}\left[y_{t}-x_{t}^{\prime }\theta_{B}-x_{t}^{\prime }\delta 1\right.\\ & \times {\left.\left\{f_{t}^{\prime }\tau >0\right\}\right]}^{2}, \end{array}$$

where *δ* = *θ*_*A*_ − *θ*_*B*_ . Instead of multidimensional grid search over *τ* , Lee et al. (2018) propose an equivalent optimization problem by introducing a set of binary parameters $d_{t}:=1\left\{f_{t}^{\prime }\gamma >0\right\}$ and ℓ_*j*, *t*_ = *δ*_*j*_ *d*_*t*_ for *j* = 1,…, *d*_*x*_ , where *d*_*x*_ is the dimension of *x*_*t*_ . The new objective function can be written as

(2.4) $$\sum_{t=1}^{T}{\left[y_{t}-x_{t}\theta_{B}-\sum_{j=1}^{d_{x}}x_{j,t}\ell_{j,t}\right]}^{2}.$$

The equivalent optimization problem becomes a mixed integer programming problem with some additional constraints. The new optimization problem can be solved efficiently by the modern MIO solvers such CPLEX and GUROBI. One can solve the optimization jointly or by iterating between (*θ*_*B*_, *δ*) and the remaining parameters. The advantage of the new algorithm is that one can construct and estimate the model, where the regimes are determined in a more sophisticated way by a multidimensional factor *f*_*t*_ . We refer to Lee et al. (2018) for additional details.

By applying the joint and iterative algorithms proposed in that paper, we obtain the following results:

$$\begin{array}{cc} & \text{Joint}\;\text{algorithm}:\;\left({\hat{\tau }}_{1},{\hat{\tau }}_{0}\right)=\left(-1.82,11.97\right),\\ & \;\mathrm{obj}=0.0002636456, \end{array}$$

$$\begin{array}{cc} & \text{Iterative}\;\text{algorithm}:\;\left({\hat{\tau }}_{1},{\hat{\tau }}_{0}\right)=\left(0.56,11.97\right),\\ & \;\mathrm{obj}=0.0002636456. \end{array}$$

That is, two algorithms yield different estimates but the same objective function values. It turns out that the regimes determined by two estimates are identical; that is, ${\hat{\tau }}_{1}$ has no role in determining slack periods.

In addition, we apply the model selection algorithm proposed in our previous work (Lee et al. 2018). Specifically, we specify the penalized least squares objective function with the penalty term consisting of a tuning parameter *λ* > 0 times the number of nonzero coefficients. The resulting specification of the endogenous sample splitting rule is as follows:

$$\sum_{t=1}^{T}{\left[y_{t}-x_{t}\theta_{B}-\sum_{j=1}^{d_{x}}x_{j,t}\ell_{j,t}\right]}^{2}+\lambda {\left|\tau \right|}_{0},$$

where |·|_0_ is an ℓ_0_ norm of a vector. We implement it using MIO with $\lambda ={\hat{\sigma }}^{2}\log\left(T\right)\text{/}T$, where *T* is the sample size and ${\hat{\sigma }}^{2}=0.00027$ is estimated from the baseline model with a single threshold at 11.97%. When we apply the penalized estimation algorithm, we find that the *τ*_1_ estimate becomes zero and is dropped from the model. Therefore, there is no empirical evidence that supports time‐varying regimes of slackness.

## III CONCLUSIONS

We have investigated state‐dependent effects of fiscal multipliers and have found that it is crucial how to determine whether the U.S. economy is in a slack state. When the slack state is defined as the period of the unemployment rate higher than about 12%, the estimated cumulative multipliers are significantly larger during slack periods than nonslack periods and are above unity. Our estimation results emphasize the importance of endogenous sample splitting. Furthermore, the effect of the fiscal policy may be heterogenous with respect to the level of slackness in the economy, thereby calling for more research in understanding the heterogenous effects of fiscal policy. Finally, our paper sheds light on the prospect of fiscal policy in response to economic shocks from the current COVID‐19 pandemic.

## Endnotes

1
*Source*: U.S. Bureau of Labor Statistics, https://www.bls.gov/news.release/empsit.nr0.htm, accessed on May 25, 2020.
